# The influence of job search stress on college students’ addictive social media use: Seeking of social support and perceived social support as serial mediators and sense of coherence as a moderator

**DOI:** 10.3389/fpsyg.2023.1101674

**Published:** 2023-02-22

**Authors:** Eric (Zeqing) Mao, Lishou Zhao

**Affiliations:** School of Cultural Creativity and Management, Communication University of Zhejiang, Hangzhou, China

**Keywords:** job search stress, social support, social media use, sense of coherence, college students

## Abstract

Resulting from an enlarged number of graduating college students and shrinkage of work opportunities, stress in relation to job search and employment is becoming an increasingly noticeable issue in China. Previous psychiatry research has suggested that social support can be conducive to reducing stress from multiple sources, while the effectiveness hinges on whether it is actually recognized and perceived by the recipients. The prevalence of social media has greatly facilitated the communication and exchange of social support information. However, they can also lead to overuse and addiction problems. This study aims to investigate how job search stress affects graduating college students’ social media addiction severity using a serial mediation model and test the potential moderation effect of sense of coherence. Based on a sample of graduating college students (*n* = 144), our findings point out a significant pathway for the impacts of stress sequentially through seeking of social support and perceived social support. Furthermore, job search stress seems to have pronounced effects on the psychological need for social support only at low- and mid-levels of sense of coherence.

## 1. Introduction

Chinese college students are now facing unprecedentedly high difficulty in finding a job and getting employed with a record 10.8 million graduates poised to enter the workforce (Ren, 2022). In addition to the negative impacts of COVID-19 (Kaye et al., 2021; Ke and Hsiao, 2022), a number of concurrent factors, such as property market downturn and regulatory crackdowns on education, have greatly decelerated China’s economic growth, making the competition for jobs even more fierce (Pollard, 2022). Consequently, in the post-pandemic era, Chinese college students have become extraordinarily vulnerable to stressors associated with job search as well as the fear of failing to secure employment. Existing research on youth psychology points out that unemployment at one’s twenties can easily cause poor mental health status, including distress, anxiety, depression, and low self-efficacy (Winefield and Tiggemann, 1985; Hammarström, 1994; Schaufeli, 1997; Parola and Marcionetti, 2022), and undesirable behavioral outcomes such as alcohol, cigarette, and drug abuse (Hammarström, 1994; Katon et al., 2007; Reissner et al., 2011).

Among many coping strategies to deal with stressful and emotionally negative experiences, seeking of social support deserves increasing attention as the ubiquitous use of social media have substantially facilitated the process of support exchange. Social support refers to “an exchange of resources between two individuals perceived by the provider or the recipient to be intended to enhance the well-being of the recipient” (Shumaker and Brownell, 1984, p. 11). Research has demonstrated that social support seekers can capitalize on social media platforms, such as Facebook and Twitter, to alleviate depressed mood by receiving response messages from known and unknown people (Rui et al., 2013; Davis et al., 2015; Lisitsa et al., 2020). That being said, it is worth noting that the effectiveness of social support hinges on whether the sense of being supported/cared for is de facto recognized and perceived by the recipient (Frison and Eggermont, 2015; Çamaş and Yalçin, 2021). Meanwhile, provided the benefits of leveraging social media to lubricate online social support exchange, there is growing concern about the related risks of excessive engagement in social media (Tang et al., 2016; Brailovskaia et al., 2019). Researchers have found overuse of social media can be detrimental to work performance, social relationships, sleep quality, and life satisfaction (Koc and Gulyagci, 2013; Kuss et al., 2014; Fox and Moreland, 2015; Sun and Zhang, 2021), in addition to cultivating feelings of jealousy, depression, and anxiety (Elphinston and Noller, 2011; Pantic, 2014). It is established in the psychiatry literature that behavioral indicators of addictive social media use (SMU) entail salience, mood modification, tolerance, withdrawal symptoms, conflict, and relapse (see D’Arienzo et al., 2019).

A variety of risk factors for problematic SMU have been well identified and examined (Wu et al., 2013; Yu et al., 2016; Dailey et al., 2020; Peris et al., 2020), including personality traits (e.g., extraversion, shyness, and self-esteem), cognitive functions (e.g., outcome expectancies and self-efficacy), and psychosocial well-being (e.g., optimism and loneliness). Aside from individual characteristics, there exist salient environmental stressors likely to trigger patterns of maladaptive use of social media. For example, Mahamid and Berte (2019) study focused on geopolitical vulnerability revealed that young adults residing in military occupied territories had a significantly higher chance to develop problematic SMU. In a similar vein, He et al. (2021) found low family socioeconomic status to be a reliable predictor of addictive SMU among female college students in China. Considering the grossly increased difficulty in finding a job and getting employed (Gu and Mao, 2023), it is not uncommon to see Chinese job seekers express dissatisfaction, publicly or privately, on many social media applications, in attempt to empathize with others in a comparable circumstance. To our best knowledge, however, limited research has investigated how job search stress impact job seekers’ problematic SMU propensity.

To fill this gap, the current study proposed and examined a behavioral model wherein the effects of stress resulting from the process of job search on the likelihood of developing problematic SMU are serially mediated by the intention to seek social support and the degree to which social support is perceived. Graduating college students devoted to job search were invited to participate in an online survey to evaluate how much stress they felt and how much social support they perceived from using WeChat Moments (also known as “Friends’ Circle”), a platform where users can launch intimate and private communications within their choice of close friends by posting texts, images, and videos (Wang et al., 2018; Zheng et al., 2022).

In the relevant literature, it is well documented that stress associated with job search and unemployment can increase job seekers’ psychological need for social support (Vinokur and Caplan, 1987; Lim, 1996; Ślebarska et al., 2009). Dunn and O’Brien (2009) revealed that a considerable fraction of Latina/Latino immigrants in the United States were severely plagued by either low paying jobs or a dearth of job opportunities. Their study found that social support from family members and significant others can help ease stress from such sources. On the other hand, while perceived social support has been observed to be an important contributory factor to the habitual use of social media (McDougall et al., 2016; Lu and Hampton, 2017), there is a body of research reporting that online social support exchange is closely aligned with problematic SMU (Tang et al., 2016; Brailovskaia et al., 2019; Liu and Ma, 2019). Drawn from attachment theory, Liu and Ma (2019) demonstrated that online social support mediates the relationship between avoidant attachment and SNS addiction based on a sample of college students in China. With this in mind, we propose three related hypotheses as follows:

*Hypothesis 1* (H1). Seeking of social support will mediate the relation between job search stress and problematic SMU.

*Hypothesis 2* (H2). Perceived social support will mediate the relation between seeking of social support and problematic SMU.

*Hypothesis 3* (H3). Seeking of social support and perceived social support will sequentially mediate the relation between job search stress and problematic SMU.

In addition, we incorporated sense of coherence (SOC) as a potential moderator, considering its inherent links with stress and social support (Wolff and Ratner, 1999; Amirkhan and Greaves, 2003; Langeland and Wahl, 2009; Schmuck et al., 2021) as well as those with substance and behavioral addictions (Feigin and Sapir, 2005; Arévalo et al., 2008; Yang et al., 2021). Specifically, SOC was developed by Antonovsky (1987) as the core tenets of his salutogenic model, which emphasizes the origin of health rather than identifying the mechanisms that lead to illness (i.e., pathogenesis). SOC is composed of three integral components: comprehensibility, manageability, and meaningfulness. According to the definitions provided by Olsson et al. (2006).

Comprehensibility refers to whether or not inner and outer stimuli make sense to us in terms of being coherent, ordered, cohesive, structured and clear. Manageability refers to the extent to which we feel resources are at our disposal to help meet the demands posed by the stimuli to which we are exposed. Meaningfulness refers to whether we can perceive life’s difficulties as ‘welcomed’ challenges worthy of an investment of energy, engagement and dedication rather than as a burden that we would prefer to avoid (p. 219).

In particular, a high SOC score often indicates high levels of trait resilience and self-efficacy (Posadzki et al., 2010; Konaszewski et al., 2021), both of which serve as critical coping resources (Cicognani, 2011). To some extent, individuals with strong SOC should be more receptive to difficulties as they typically possess an enriched collection of coping strategies (Cohen and Dekel, 2000). Hence, they should have greater ability to manage stress independently and be comparatively less reliant on seeking help from others (Çamaş and Yalçin, 2021). Accordingly, we propose another hypothesis:

*Hypothesis 4* (H4). SOC will moderate the relation between job search stress and seeking of social support.

## 2. Materials and methods

### 2.1. Participants

An online survey was conducted at a university in East China in April 2022, during which period graduating college students were usually involved in the intense process of searching jobs and seeking employment. For selecting appropriate respondents, a filter question was inserted to exclude those who had no plan to find a job at all or had already reaped satisfactory job offers considering they might over- or under-estimate stress in relation to job search. A digital information sheet, containing a brief summary of the research and its aims in addition to an invitation link, was sent out to the email addresses of 500 randomly selected senior undergraduate students in the university. While 208 responses were initially collected, 64 (30.7%) were dropped based on the selection criterion or because of invalid answers, such as filling out the age question with 120. Therefore, the final sample used for this study consisted of 144 respondents, among whom 43 (29.9%) were male and 101 (70.1%) were female. Their ages ranged from 21 to 24 years, with a mean of 22.35 (*SD* = 0.79). Our study was approved by the Institutional Review Board (IRB) at the university, and voluntary informed consent was obtained from the participants, who were granted anonymity and confidentiality.

### 2.2. Measures

#### 2.2.1. Job search stress

Following da Motta Veiga and Turban (2014), job search-related stress was measured using three items from the Explorational and Decisional Stress Scale of the Career Exploration Survey developed by Stumpf et al. (1983). With a 7-point Likert scale (1 = “Not stressful”, 7 = “Extremely stressful”), the respondents were asked to rate “how much unintended stress has the following caused compared to other important issues which you have had to contend in the last two months.” To be precise, the three items were “looking for a job,” “interviewing with specific companies,” and “deciding what work I want to do.” The Cronbach’s alpha for our sample was 0.88, indicating good internal consistency.

#### 2.2.2. Social support seeking and perceived social support

Items for measuring the respondents’ motive to seek social support and perception of social support were adapted from the work of Frison and Eggermont (2015), who operationalized the relevant constructs within the context of Facebook. In our study, items about seeking of social support through WeChat Moments included “I turn to WeChat Moments to seek help” and “I turn to WeChat Moments to talk with someone about my problems,” both starting with “If you are feeling down or in a difficult situation.” The Cronbach’s alpha was 0.94, suggesting excellent internal consistency. In regards to perceived social support, the items were “I can find help on WeChat Moments,” “I can find emotional help and support that I need on WeChat Moments,” “I can talk with someone on WeChat Moments about my problems,” and “I can find someone on WeChat Moments who helps me take decisions.” The reasons why we focused on WeChat Moments are twofold. First, WeChat, developed and operated by *Tencent*, is a national real-time social media app in China, with the Moments function being a defining feature. Second, users entitled to read one’s WeChat Moments posts are basically friends and acquaintances, who are supposed to be more likely to afford social support. The items were deemed internally consistent, with the Cronbach’s alpha being 0.82. A 5-point Likert scale was adopted, which ranged from 1 (“Strongly disagree”) to 5 (“Strongly agree”), for the above items.

#### 2.2.3. Social media addiction

For assessing the respondents’ problematic SMU severity, we utilized the Chinese Social Media Addiction Scale developed and validated by Liu and Ma (2018). The scale was characterized by six categories, including preference for online social interaction, mood alteration, negative consequence and continued use, compulsive use and withdrawal, salience, and relapse, with each having multiple items that were scored based on a 5-point Likert scale (1 = “Strongly disagree”, 5 = “Strongly agree”). The sample’s internal consistency reliabilities measured as Cronbach’s alpha for the six categories were 0.92, 0.86, 0.79, 0.86, 0.90, and 0.81, respectively.

#### 2.2.4. Sense of coherence

Sense of coherence was graded using a series of semantic differential items on a 7-point Likert scale, with high scores denoting a strong SOC. Antonovsky (1987, 1993) seminal papers have substantiated the psychometric properties of the SOC scale to be valid and reliable. In our study, we used the short form 9-item semantic differential scale (Midanik et al., 1992; Klepp et al., 2007), including items such as “Doing the things you do every day is” with answers ranging from 1 (“A source of pain and boredom”) to 7 (“A source of deep pleasure and satisfaction”). These items were considered internally consistent as the Cronbach’s alpha was 0.90.

### 2.3. Statistical analysis

Descriptive statistics and zero-order correlations of the variables analyzed in this study are displayed in Table 1. The software R was capitalized on to statistically examine the hypothesized serial mediation and moderation effects with structural equation modeling (SEM). In particular, to test the moderating effect, we classified the participants into three subgroups according to their SOC scores: low SOC (1 *SD* below the mean; *n* = 22), mid SOC (between 1 *SD* below and 1 *SD* above the mean; *n* = 98), and high SOC (1 *SD* above the mean; *n* = 24). The interaction effect of three levels of SOC and the intent to seek social support was visualized using a Johnson-Neyman plot. We evaluated the fitness of model based on comparative fit index (CFI), incremental fit index (IFI), and root-mean squared error of approximation (RMSEA). To be more precise, CFI and IFI values greater than 0.95 (Bollen, 1989; Bentler, 1990) and RMSEA values smaller than 0.05 (Kline, 2004) were adopted as acceptable cut-offs for the fitness indices.

**TABLE 1:** Descriptive statistics and zero-order correlations of the variables analyzed in this study.

Variable	*M* (*SD*)	1	2	3	4	5	6	7	8	9	10	11
Job Search Stress	3.83 (0.92)	-										
Seeking of Social Support	3.12 (0.99)	0.32***	-									
Perceived Social Support	3.07 (0.92)	0.33***	0.72***	-								
Social Media Addiction-Preference for Online Social Interaction	3.08 (0.99)	0.35***	0.78***	0.75***	-							
Social Media Addiction-Mood Alteration	3.08 (0.98)	0.18*	0.31***	0.15	0.26**	-						
Social Media Addiction-Negative Consequence and Continued Use	3.19 (1.32)	0.06	0.08	-0.08	0.07	0.67***	-					
Social Media Addiction-Compulsive Use and Withdrawal	2.99 (0.83)	0.23**	0.62***	0.59***	0.59***	0.52***	0.17*	-				
Social Media Addiction-Salience	3.07 (0.82)	0.26**	0.57***	0.52***	0.51***	0.56***	0.16*	0.89***	-			
Social Media Addiction-Relapse	3.02 (0.83)	0.24**	0.59***	0.57***	0.58***	0.54***	0.17*	0.96***	0.85***	-		
Sense of Coherence	3.34 (1.08)	-0.26**	-0.23**	-0.10	0.03	0.07	0.09	-0.10	-0.07	-0.12	-	
Age	22.35 (0.79)	-0.09	0.01	0.04	0.02	0.04	-0.02	0.07	0.08	0.06	-0.03	–
Sex	–	0.36***	0.15	0.32***	0.25**	0.13	0.09	0.24**	0.19*	0.20*	0.11	0.02

*p < 0.05, **p < 0.01, ***p < 0.001.

## 3. Results

### 3.1. Descriptive statistics

Table 1 presents the descriptive statistics of the variables analyzed in this study, including means, standard deviations, as well as zero-order correlations. The descriptive results indicate that job search stress was positively associated with the intent to seek social support (*r* = 0.32) and the perception of social support (*r* = 0.33) at the 0.1% significance level, whereas it was moderately and positively correlated with the social media addiction symptoms except negative consequence and continued use (*r* = 0.06, *p* > 0.05). Seeking of social support and perceived social support were significantly and positively correlated (*r* = 0.72, *p* < 0.001), and they were found to have positive relations with most of the addictive SMU symptoms. Concerning the moderator SOC, it was negatively linked with job search stress (*r* = −0.26, *p* < 0.01) and seeking of social support (*r* = −0.22, *p* < 0.01). No significant correlations were observed between SOC and other variables of interest. As to the control variables, age was uncorrelated with any of the other variables, probably owing to its limited variation (*CV* = 0.04). On the contrary, there were significant gender differences (male serving as the reference) with respect to job search stress (*r* = 0.36, *p* < 0.001) and perceived social support (*r* = 0.32, *p* < 0.001), as well as some of the addictive SMU symptoms. As such, only gender was included as a control variable in the following analyses.

### 3.2. Serial mediation analysis and moderation effect

**FIGURE 1:**
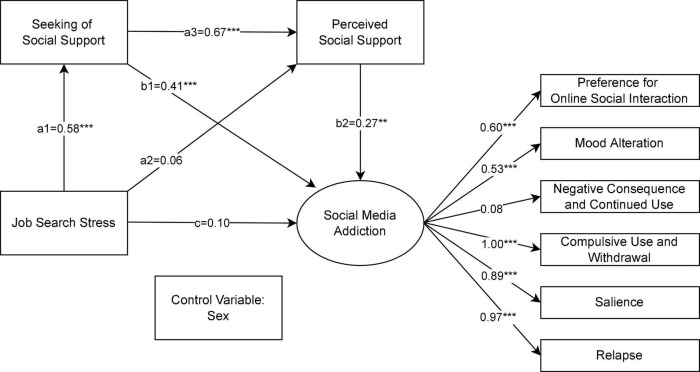
Structural equation model denoting the relation between job search stress and social media addiction after controlling for gender. Standardized coefficients are reported. *p < 0.05, **p < 0.01, ***p < 0.001.

The results of serial mediation analysis on the relation between job search stress and problematic SMU, mediated sequentially through seeking of social support and perceived social support with gender being controlled for, are illustrated in Figure 1. It was detected that the intent to seek social support, namely the first proposed mediator of the relationship between job search stress and problematic SMU, was significant (a_1_b_1_ = 0.24, *p* < 0.05). Thus, H1 was supported. Differently, the second predicted mediator, i.e., perceived social support, was found to be not significant (a_2_b_2_ = 0.02, *p* > 0.05). Accordingly, H2 was rejected. The serial indirect effect, which passed in sequence through seeking of social support and perceived social support, was observed to be significantly positive (a_1_a_3_b_2_ = 0.11, *p* < 0.05), supporting H3. Although job search stress did not directly influence problematic SMU (c = 0.10, *p* > 0.05), the total effect of job search stress on problematic SMU was statistically significantly (a_1_b_1_ + a_2_b_2_ + a_1_a_3_b_2_ + c = 0.47, *p* < 0.05). In sum, our findings suggested that job search stress affected problematic SMU mainly through two pathways: (1) being mediated through the intent to seek social support; and (2) being mediated sequentially through seeking of social support and perceived social support. The indices of model fit were CFI = 0.92, TLI = 0.90, and RMSEA = 0.04, respectively, indicating good fitness of the specified mediation model.

**FIGURE 2:**
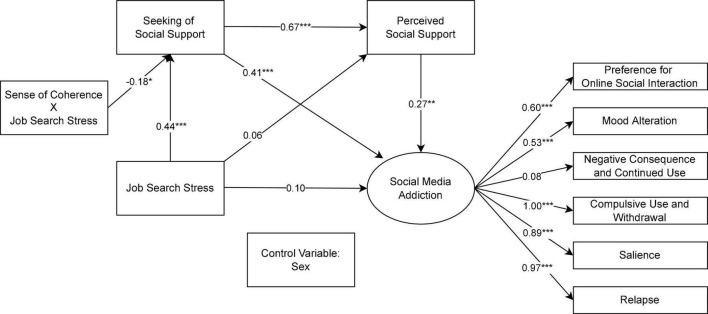
Structural equation model denoting the relation between job search stress and social media addiction with sense of coherence as the moderator after controlling for gender. Standardized coefficients are reported. *p < 0.05, **p < 0.01, ***p < 0.001.

**FIGURE 3:**
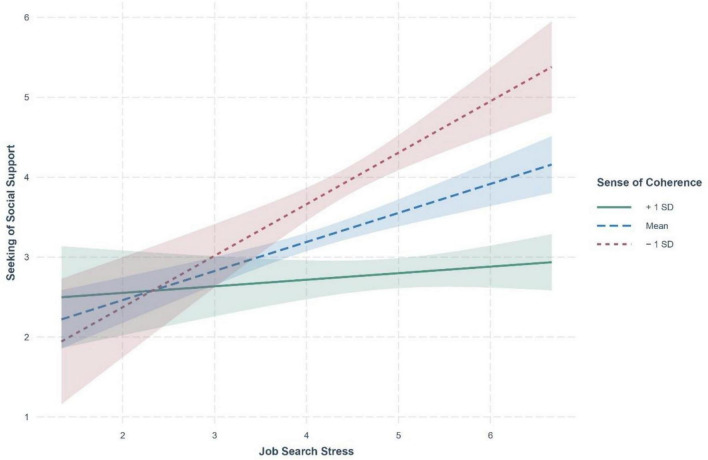
Johnson-Neyman plot presenting the interaction effect between job search stress and sense of coherence.

Figure 2 reports the moderation effect of SOC on the linkage between job search stress and seeking of social support. As aforementioned, we stratified the sample into three subgroups according to the level of SOC and then reexamined the relationship in question. Notably, the interaction term of job search stress and SOC negatively predicted the intent to seek social support (β = −0.18, *p* < 0.05), meaning that the inclusion of SOC altered the way the psychological need for social support reacted to increased job search stress. Figure 3 presents a Johnson-Neyman plot, delineating the cross-over interaction effects of job search stress and low (1 *SD* below mean; *n* = 22), mid (between –1 to 1 *SD*; *n* = 98), and high (1 *SD* above mean; *n* = 24) levels of SOC on seeking of social support. It can be seen that the positive association between the two variables under consideration was only pronounced at low and mid SOC (β = 0.62, *p* < 0.001; β = 0.36, *p* < 0.001), whereas job search stress marginally predicted the intent to seek social support at high SOC (β = 0.08, *p* > 0.05). As such, the significantly positive impact of job search stress on seeking of social support observed in the previous serial mediation model appeared to depend on the level of SOC, which supported H4.

## 4. Discussion

The aim of our study was to investigate how stress resulting from job search impact graduating college students’ addictive SMU severity, as serially mediated by seeking of social support and perceived social support, in addition to the potential moderation effect of SOC on the relationship between job search stress and seeking of social support. The results obtained in this study supported H1 that the psychological need for social support will mediate the linkage between job search stress and problematic SMU but rejected H2 that perceived social support will mediate the relation between these two variables. Moreover, the results pertinent to H3 pointed out a significant pathway sequentially through seeking of social support and perceived social support. These findings resonated with the extant literature that individuals suffering from stress are more inclined to seek social support (Taylor et al., 2004; Chib et al., 2013). This is primarily because social support can make people believe they are “cared for, loved, esteemed, and a member of a network of mutual obligations” (Cobb, 1976, p. 300), and accordingly supportive interactions with others can help prevent undesired consequences of life stress (Cohen and Wills, 1985). Meanwhile, however, increased engagement in seeking social support in turn can readily trigger addiction to the use of social media (Tang et al., 2016; Bilgin and Taş, 2018; Alimoradi et al., 2019), which functions as a pivotal platform in today’s society for communicating and exchanging social support information. Therefore, it is warranted to expand the theoretical and practical understanding of how such a well-intentioned behavior can ultimately lead up to unintended outcomes (or side effects).

With regards to H4, the moderating role of SOC on the relationship between job search stress and seeking of social support was clearly demonstrated as more in-depth analysis showed the impact was only prominent at low- and mid-levels of SOC. Such a result is consistent with previous research (Wolff and Ratner, 1999; Amirkhan and Greaves, 2003) in that people with a strong SOC are predisposed to perceive life events as less stressful (i.e., comprehensibility), possess the ability to search and mobilize resources to handle encountered stressors (i.e., manageability), and have the inherent motivation and commitment to materialize coping strategies (i.e., meaningfulness). This can help rationalize the finding that the effect of job search stress on seeking of social support became insignificant in the high SOC case. Our study makes an empirical contribution to the broader discussion surrounding how personal trait (e.g., SOC) can moderate the impact of situational stimuli (e.g., stress) on behavioral tendencies (e.g., seeking of social support).

Provided some interesting findings, our study has several limitations that might restrain its contributions to the literature. First, our study is essentially cross-sectional, analyzing data collected at one given point in time across the population of graduating college student. This research design is unable to capture how the variables of interest change over short or long periods of time. Therefore, future research on this topic can consider undertaking longitudinal studies to address the drawback. Second, our study did not distinguish different motivations to seek social support, though the relevant literature has categorized social support into four types: appraisal, instrumental, informational, and emotional (Olson and Shultz, 1994). Third, we only investigated a certain type of social media, i.e., WeChat Moments, given that there also exist other popular Chinese social media apps and platforms, such as Weibo and Douban, where users can also freely exchange social support information. Last but not least, our sample was largely restricted to college students in higher education institutions. However, another group of graduating students needs to be addressed is those of vocational schools, who are faced with such a harsh environment of job search and even more adverse conditions due to the society’s discrimination against them. Future research may consider taking into account a greater diversity of graduating students who have pressure on securing a job.

## 5. Conclusion

This study revealed that job search stress can indirectly impact the severity of job seekers’ addiction to social media use, mediated by the psychological need for social support or serially through seeking of social support and perceived social support. Furthermore, sense of coherence was found to be an important moderator of the relation between job search stress and seeking of social support. Individuals with a strong sense of coherence tend to be less reliant on social support as a coping means to mitigate stress related to job search.

## Data availability statement

The raw data supporting the conclusions of this article will be made available by the authors, without undue reservation.

## Ethics statement

The studies involving human participants were reviewed and approved by the Institutional Review Board of School of Cultural Creativity and Management, Communication University of Zhejiang. The patients/participants provided their written informed consent to participate in this study.

## Author contributions

EM: study conception and design, data collection, and draft manuscript preparation. EM and LZ: analysis and interpretation of results. Both authors reviewed the results and approved the final version of the manuscript.
